# The Rieske iron-sulfur protein is a primary target of molecular hydrogen

**DOI:** 10.1016/j.redox.2025.103952

**Published:** 2025-11-27

**Authors:** Shuto Negishi, Mikako Ito, Tomoya Hasegawa, Hikaru Otake, Bisei Ohkawara, Akio Masuda, Hiroyuki Mino, Tyler W. LeBaron, Kinji Ohno

**Affiliations:** a Division of Neurogenetics, Center for Neurological Diseases and Cancer, Nagoya University Graduate School of Medicine, Nagoya, 466-8550, Japan; b Division of Material Science (Physics), Nagoya University Graduate School of Science, Nagoya, 464-8601, Japan; c Department of Kinesiology and Outdoor Recreation, Southern Utah University, Cedar City, UT, 84720, USA; d Molecular Hydrogen Institute, Enoch, UT, 84721, USA; e Graduate School of Nutritional Sciences, Nagoya University of Arts and Sciences, Nisshin, 470-0196, Japan

**Keywords:** Molecular hydrogen, Rieske iron-sulfur protein, Mitochondrial unfolded protein response, Hydrogenase

## Abstract

The mechanisms underlying the biomedical effects of molecular hydrogen (H_2_) remain poorly understood and are often attributed to its selective reduction of hydroxyl radicals, based on the long-held notion that H_2_ is biologically inert. We demonstrate that H_2_ is biologically active, specifically targeting the Rieske iron-sulfur protein (RISP). We first observed that H_2_ induces the mitochondrial unfolded protein response (UPR^mt^) in cultured cells exposed to H_2_ and in mouse liver after H_2_ water administration. H_2_ suppressed electron transport chain complex III activity in mouse liver homogenates to 78.5 % within 2 min. Given the evolutionary link with hydrogenases, we examined RISP as a potential target of H_2_. We found that H_2_ promotes RISP degradation within 1 h in cultured cells by activating mitochondrial Lon peptidase 1 (LONP1). Loss of RISP and subsequent UPR^mt^ induction may explain the pleiotropic and paradoxical effects of H_2_. These findings identify RISP as a primary target of H_2_, demonstrating that H_2_ is biologically active as a signaling molecule.

## Graphical abstract

**Figure:**
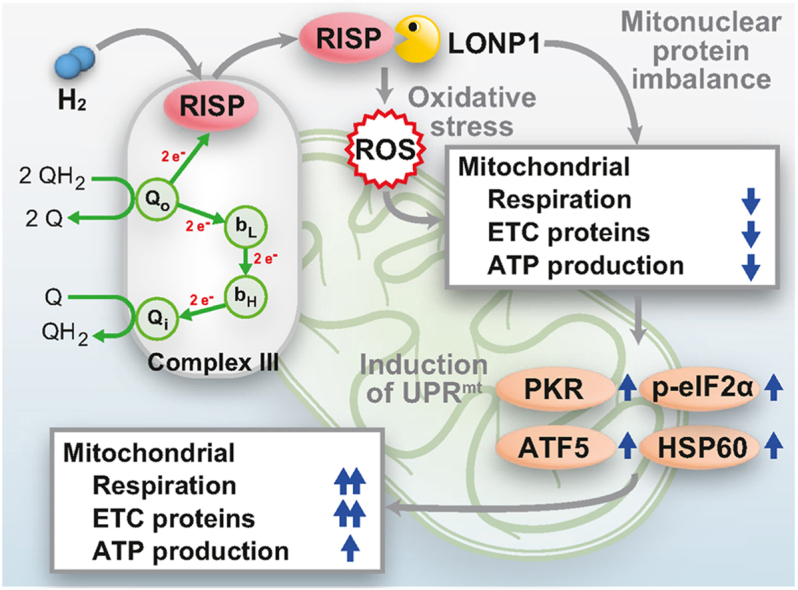
H_2_ triggers LONP1-mediated degradation of the Rieske iron-sulfur protein (RISP) and initially suppresses mitochondrial ETC activity, which subsequently induces mitochondrial unfolded protein response (^mt^UPR) and activates mitochondrial ETC activity.

## Abbreviations

ETC: Electron transport chain
LONP1: Mitochondrial Lon peptidase 1
RISP: Rieske iron-sulfur protein
ROS: Reactive oxygen species
UPR^mt^: Mitochondrial unfolded protein response

## 1 Introduction

The therapeutic potential of molecular hydrogen (H_2_) dates back to 1793 and its reaction kinetics with hydroxyl radicals (^•^OH) were characterized in the 1960s [1]. However, H_2_ has long been regarded as biologically inert due to its small, neutral, and nonpolar nature, compounded by the absence of hydrogenase enzymes in humans. Unlike other signaling molecules such as the gasotransmitters, carbon monoxide (CO), hydrogen sulfide (H_2_S), and nitric oxide (NO^•^), H_2_ was presumed to lack the physicochemical properties necessary to interact with biological macromolecules. This presumption as a biologically inert gas justified its use as a safe, inert breathing gas for deep-sea diving since the 1940s [2] and relegated its role in physiology to that of a mere microbial byproduct [3].

Emerging evidence, however, suggests that increased endogenous H_2_ production by the microbiome is associated with improved metabolic and cognitive health, reduced cardiovascular risk, and extended lifespan in centenarians [4]. However, no cogent mechanism has been provided to explain these important observations.

A pivotal shift in the perception of H_2_ occurred in 1975, when a *Science* publication reported that hyperbaric hydrogen, but not helium led to the marked regression of tumors in mice, attributed to its presumed ability to scavenge cytotoxic ^•^OH radicals [5]. This mechanistic premise resurfaced more prominently in 2007 by a *Nature Medicine* study demonstrating that H_2_ ameliorated brain damage in a rat stroke model [6]. Both studies relied on the radical scavenging mechanism. However, this concept is challenged by H_2_'s slow second-order reaction rates relative to other more abundant nucleophilic biomolecules [7]. Nevertheless, research into H_2_ has proliferated, with over 3000 publications, including 200 human studies highlighting its potential therapeutic effects [8]. Yet, the primary molecular target(s) and mechanisms of H_2_'s activity have remained elusive, with much of the literature still framing H_2_ as merely an antioxidant. Most recently, it was demonstrated that H_2_ can bind to Fe-porphyrin and the hydrided Fe-porphyrin reduces hydroxyl radicals [9]. In addition, CO_2_ bound to Fe-porphyrin is changed to CO by H_2_. However, the amounts of reduced hydroxyl radicals and the generated CO may be too low to exert efficient biological effects. Similarly, the concentrations of H_2_ are too low and the dwell time of H_2_ is too short *in cellulo* and *in vivo* [8,10] to account for the prolonged (i.e., hours to days) residual protective effects of H_2_.

Given the limitations of the current proposed mechanisms, we hypothesized that H_2_ might interact with an evolutionarily conserved hydrogenase-like protein harboring iron-sulfur [Fe–S] clusters. The Rieske iron–sulfur protein (RISP), encoded by *UQCRFS1*, in Complex III of the mitochondrial electron transport chain (ETC) carries an [2Fe–2S] cluster and is a compelling candidate for interaction with H_2_. RISP shuttles electrons from ubiquinol to cytochrome *c*_*1*_ within the Q-cycle. Although RISP lacks a canonical H_2_ activation or binding site, its conserved [2Fe–2S] cluster shares structural and evolutionary similarities with hydrogenase catalytic centers.

Mitochondrial dysfunction activates the mitochondrial unfolded protein response (UPR^mt^), an adaptive pathway that maintains mitochondrial homeostasis through mitohormetic mechanisms [11]. Sobue et al. reported that H_2_ induced UPR^mt^ in the mouse liver [12], and we previously showed that H_2_ improved mitochondrial function in cells in which H_2_ induced UPR^mt^ [13].

Here, we show that H_2_ directly targets RISP, leading to its LONP1-mediated degradation, suppressing Complex III activity, and subsequently inducing UPR^mt^. This targeted effect illuminates a previously unrecognized mechanism, redefining H_2_ from a biologically inert molecule to a biologically active signaling molecule that modulates mitochondrial signaling pathways.

## 2 Materials and methods

### 2.1 Animal studies and *ad libitum* administration of H_2_ water

All animal studies were approved by the Animal Care and Use Committee of the Nagoya University, and were conducted in accordance with relevant guidelines. Seven-week-old C57BL6/N mice were purchased from Japan SLC. H_2_ water was freshly prepared every evening using Hydrogen Water 7.0 (Ecomo International), which was kindly provided by MiZ Co. Ltd. The H_2_ concentrations in freshly prepared water were 2.5–3.5 mM. The H_2_ concentration in the glass vessel that was inserted in the mouse cage decreased exponentially with a half-life of 1.09 h [35]. As mice drink water approximately every hour at night, mice were predicted to drink H_2_ water with an average concentration of 1.7 mg/L [35]. At 7 weeks of age, mice started drinking H_2_ water. After 4 weeks, mice were fasted for 17 h and were sacrificed under deep anesthesia with isoflurane. Whole liver tissue was collected and rapidly frozen in liquid nitrogen.

### 2.2 Cell culture and exposure to H_2_ gas

We previously analyzed the effects of H_2_ on AML12, A549, HCT116, HeLa, HepG2, HT1080, PC3, and SH-SY5Y cells, and found that the first five cells (AML12, A549, HCT116, HeLa, and PC3 cells) were more responsive to H_2_ than the other cells [13]. As the four cell lines other than AML12 cells were cancer cells, we used AML12 cells that were derived from normal mouse hepatocytes. AML12 cells were purchased from ATCC. The cells were cultured in the DMEM/F-12 medium (Gibco) containing 10 % fetal bovine serum (FBS, Thermo Fisher Scientific), dexamethasone (Sigma), and insulin-transferrin-sodium selenite (Sigma). Six- or 96-well culture plates were placed in a 560-ml closed plastic box that was humidified with water at the base of the box. The box was placed in an incubator (SLI-221, EYELA) and the air temperate inside the box was maintained at 37 °C. H_2_ or N_2_ gas (6 mL/min) was mixed with CO_2_-added air (5 % CO_2_ and 95 % air, 54 mL/min) to make 10 % H_2_ or 10 % N_2_ gas. As the air contains 78.1 % N_2_, the N_2_ concentration in the gas mixture labeled as 10 % N_2_ gas is 76.8 %, but for simplicity it is referred to as 10 % N_2_ gas in this communication. The mixed gas was introduced into the box via an afferent tube, and the box was equipped with an efferent tube to expel the gas outside the room. The concentration of H_2_ in the medium was measured by equilibrating 1 mL of the medium with 100 mL of 100 % N_2_ gas in an aluminum bag. Subsequently 1 mL of the equilibrated gas was analyzed via gas chromatography (EAGanalyzer GS-23). The cellular studies were performed in triplicate or quadruplicate on the same day, and the number of dishes is indicated in each figure legend. AML12 cells were treated with 2 mM N-acetylcysteine (NAC) for 18 h prior to exposure to 10 % H_2_ for 1 h to examine whether reduction of reactive oxygen species (ROS) by H_2_ mediates the reduction of RISP.

### 2.3 Measurements of mitochondrial ETC activities in mouse liver homogenates

ETC activities were measured as previously described [36]. Briefly, 5 μL of the mouse liver homogenates or cell lysates were used for the reaction. The protein concentration of each sample was measured by the Pierce 660 nm protein assay reagent (Thermo Fisher Scientific). The activities of ETC complexes I, III, and IV were determined by the decrease in absorbance of NADH at 340 nm in 180 s, the increase in absorbance of reduced cytochrome *c* at 550 nm in 120 s, and the decrease in absorbance of reduced cytochrome *c* at 550 nm in 180 s, respectively, with NanoDrop 2000C (Thermo Fisher Scientific). We followed the incubation times in the previous report [36], and were not modulated in our assays. H_2_ was dissolved in the reaction buffer using Hydrogen Water 7.0 (2.5–3.5 mM) immediately before the homogenates were added.

### 2.4 Inhibitors of ETC complex III

Antimycin A and myxothiazol were purchased from Sigma Aldrich. Variable concentrations of the chemicals were added to the culture medium for 12 h before harvesting cells.

### 2.5 Measurements of mitochondrial superoxide level and mitochondrial membrane potential

To evaluate the acute effects of H_2_ on mitochondrial superoxide level and mitochondrial membrane potential in 10 and 30 min, H_2_ was dissolved in the culture medium using Hydrogen Water 7.0 (2.5–3.5 mM) and was added to AML12 cells. To evaluate the delayed effects of H_2_ over a period of 1–24 h, AML12 cells were cultured in an atmosphere of 10 % H_2_ or 10 % N_2_ gas as described above. After the cells were washed with PBS, the cells were incubated with either 5 μL MitoSOX (M36008, Thermo Fisher Scientific) in Hank's balanced salt solution (HBSS, Gibco) or 100 nM tetramethylrhodamine (TMRM, T668, Thermo Fisher Scientific) in PBS at 37 °C for 30 min in an incubator. The cells were harvested using 0.25 % trypsin/0.1 % EDTA in PBS and centrifuged at 3000×*g* at 4 °C for 2 min. After washing with PBS, the signal intensities of MitoSOX and TMRM were quantified using a BD FACS Calibur (BD Science).

### 2.*6* Measurement of HSPD1 (HSP60) promoter activity by luciferase reporter assay

A 1333-bp segment of human *HSPD1* promoter (positions 197,499,187 to 197,500,519 according to GRCh38) was PCR-amplified and cloned into the pGL4.10 luciferase reporter plasmid (Promega). AML12 cells were transfected with pGL4.10-*HSPD1* and phRL-TK (Renilla luciferase plasmid, Promega) using Lipofectamine 2000 (Invitrogen) according to the manufacturer's protocols. Six hours post-transfection, cells were exposed to 10 % H_2_ or 10 % N_2_ gas for 18 h. Luciferase fluorescence was measured using the Dual Luciferase Reporter Assay System (Promega) with a PowerScan4 (DS Pharma Biomedical).

### 2.7 Lonp1 knockdown and inhibition of LONP1 by CDDO-Me

siRNAs against mouse *Lonp1* were designed using the *i*-Score Designer [37]. The siRNA sequences were si783 (5′-GGUGGAGGUUGAGAAUGUA-3′) and si1194 (5′- GGAGAAAGAUGAUAAAGAU-3′). For *Lonp1* knockdown, AML12 cells were transfected with 150 pmol *Lonp1*-targeting siRNA (si783 or si1194) or control siRNA (AllStars Neg. Control siRNA, Qiagen) using Lipofectamine RNAiMax (Invitrogen) according to the manufacturer's protocols. At 48 h post-transfection, the cells were exposed to 10 % H_2_ or 10 % N_2_ gas for 1 or 12 h. To chemically inhibit LONP1, AML12 cells were treated with 0.1 or 1 μM CDDO-Me (Sigma) and cultured in 10 % H_2_ or 10 % N_2_ gas for 1 h.

### 2.8 Preparation of cell lysates and Western blotting

Cells were harvested using PLC buffer containing 50 mM HEPES (pH 7.0), 150 mM NaCl, 10 % glycerol, 1 % TritonX-100, 1.5 mM MgCl_2_, 1 mM EGTA, 100 mM NaF, 10 mM sodium pyrophosphate, 1 μg/μL aprotinin, 1 μg/μL leupeptin, 1 μg/μL pepstatin A, 1 mM PMSF, 1 mM sodium orthovanadate, and the Phosphatase Inhibitor Cocktail (PhosSTOP, Roche). The lysates were mixed on a rotary shaker at 4 °C for 15 min and centrifuged at 17,900×*g* at 4 °C for 15 min. The supernatants were boiled at 95 °C for 5 min in 2 × Laemmli buffer. Samples were then loaded on a 10 % or 14 % SDS-polyacrylamide gel, and transferred to an Immobilon-P membrane (Millipore). Membranes were washed in Tris-buffered saline with 0.05 % Tween 20 (TBS-T), and blocked with 5 % skim milk in TBS-T at room temperature for 1 h. The membranes were incubated with primary antibodies (Supplementary Table S1) overnight at 4 °C. After washing with TBS-T, the membranes were incubated with secondary goat anti-mouse IgG (1: 5000, LNA931V/AG, GE Healthcare) or anti-rabbit IgG (1: 5000, LNA934V/AE, GE Healthcare) antibody conjugated to horseradish peroxidase (HRP) for 1 h at room temperature. The antibody-bound proteins were visualized using Amersham ECL Western blotting detection reagents (GE Healthcare), and the signal intensities were quantified using ImageQuant (GE Healthcare).

### 2.9 ATP quantification assay

AML12 cells were incubated in a 10 % N_2_ gas atmosphere for 24 h before hydrogen exposure. The cells were then cultured in either 10 % H_2_ or 10 % N_2_ gas atmosphere for 1–24 h. The amount of ATP was quantified using Luminescent ATP Detection Assay Kit (ab113849, abcam).

### 2.10 Statistical analysis

All values were presented as the mean ± SEM. For *in cellulo* studies, values were normalized to those of cells treated with 10 % N_2_ gas, unless otherwise indicated. Statistical significance was assessed using Student's *t*-test, one-way ANOVA with Tukey's posthoc test, two-way ANOVA with Sidak's posthoc test, two-way ANOVA with Tukey's posthoc test, or the Jonckheere-Terpstra trend test using GraphPad Prism ver. 10.6.1 and IBM SPSS ver. 29.0.2.0. *P*-values less than 0.05 were considered statistically significant.

## 3 Results

### 3.1 H_2_ modulates mitochondrial superoxide level and mitochondrial membrane potential

**Fig. 1:**
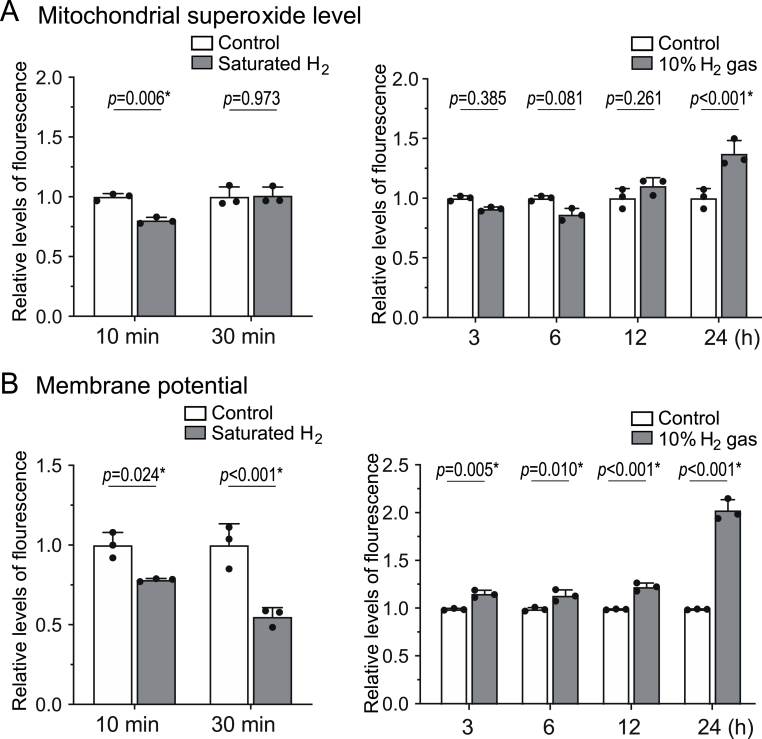
**H_2_ initially decreased the mitochondrial ETC activities and subsequently upregulates them by inducing UPR^mt^.** AML12 cells incubated under 10 % H_2_ or control gas for 10 min to 24 h were stained by MitoSOX for mitochondrial superoxide **(A)** and TMRM for mitochondrial membrane potential **(B)**. For short-time exposures (left panels), the medium was saturated with H_2_ in advance and was added to the cells. For long-term exposures (right panels), the culture plate was placed under 10 % H_2_ or control gas. *P*-values by two-way ANOVA with Sidak's posthoc test (*n* = 3 culture dishes). Statistical significance is indicated by an asterisk.

We examined the effects of H_2_ on the mitochondrial superoxide production and the mitochondrial membrane potential in AML12 cells and found that both were decreased to 80.0 % and 78.1 %, respectively, in 10 min by exposure to 10 % H_2_ gas (10 % H_2_/4.5 % CO_2_/85.5 % air) compared to control gas (10 % N_2_/4.5 % CO_2_/85.5 % air) (Fig. 1AB). However, the superoxide level and the membrane potential were increased to 116.6 % and 130.1 %, respectively, in 1 h, and to 137.2 % and 203.6 %, respectively, in 24 h. The early decrease and late increase of superoxide production and membrane potential suggest inhibition of the mitochondrial electron transport chain (ETC) and induction of mitohormesis.

### 3.2 H_2_ induces UPR^mt^ in cultured cells and wild-type mouse liver

**Fig. 2:**
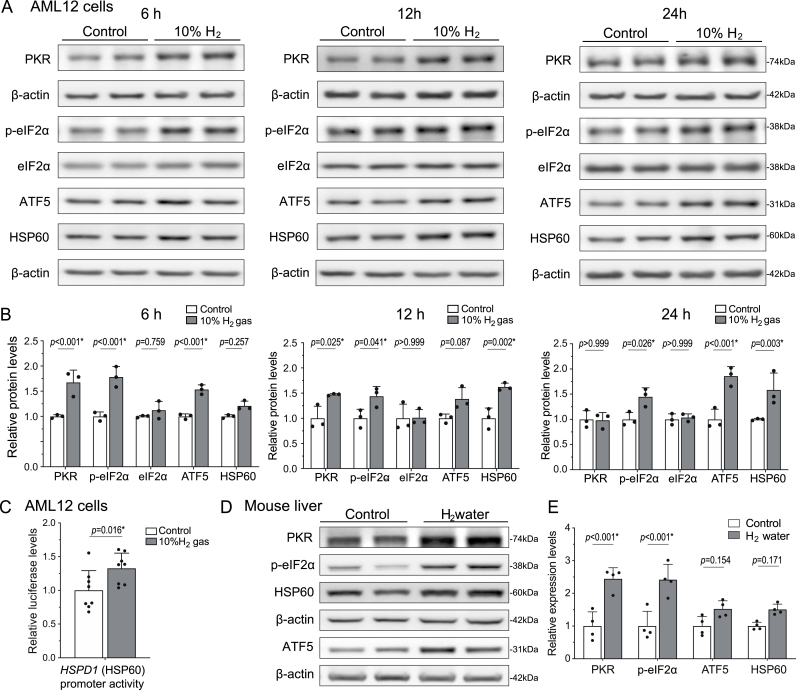
**H_2_ induced UPR^mt^ in AML12 cells and the mouse liver**. **(A, B)** AML12 cells were exposed to 10 % H_2_ or control gas for 6, 12, and 24 h. Representative Western blotting **(A)** and quantification **(B)** of UPR^mt^-related proteins (PKR, *p*-eIF2α, eif2α, ATF5, and HSP60) in AML12 cells. *P*-values by two-way ANOVA with Sidak's posthoc test are indicated (*n* = 3 culture dishes each). ∗*P* < 0.05. **(C)** AML12 cells were exposed to 10 % H_2_ or control gas for 18 h. The pGL4 luciferase activity of the human *HSPD1* (HSP60) promoter was normalized for Renilla luciferase activity (phRL-TK) and also for the ratio in control cells. *P*-value by Student's *t*-test (*n* = 8 culture dishes each). ∗*P* < 0.05. **(D, E)** C57BL6/N mice were freely accessible to H_2_-enriched water for 4 weeks. Representative Western blotting **(D)** and quantification **(E)** of UPR^mt^-related proteins (PKR, *p*-eIF2α, ATF5, and HSP60) in the mouse liver. *P*-values by two-way ANOVA with Sidak's posthoc test are indicated (*n* = 4 mice each). Statistical significance is indicated by an asterisk.

To determine the involvement of mitohormesis, we examined whether H_2_ induces UPR^mt^ in the mouse liver-derived AML12 cells and in wild-type mouse liver. Compared to control gas, exposure of AML12 cells to 10 % H_2_ gas for 6 h or longer increased the levels of UPR^mt^-related proteins (PKR, *p*-eIF2α, ATF5, and HSP60) (Fig. 2AB) and increased the promoter activity of *Hspd1* encoding HSP60 (Fig. 2C). Similarly, wild-type C57BL6/N mice that were administered H_2_-rich water *ad libitum* for four weeks exhibited elevated levels of UPR^mt^-related proteins (PKR, *p*-eIF2α, ATF5, and HSP60) in the liver, although statistical significance was not observed in ATF5 or HSP60 (Fig. 2DE). We also examined the levels of mitochondria fission- and fusion-related proteins to evaluate the effects on mitochondrial dynamics. After 6 h of H_2_ treatment, their expression levels remained unchanged in AML12 cells (Supplementary Fig. S1).

### 3.3 H_2_ reduces ATP production and suppresses the enzymatic activity of ETC complex III

**Fig. 3:**
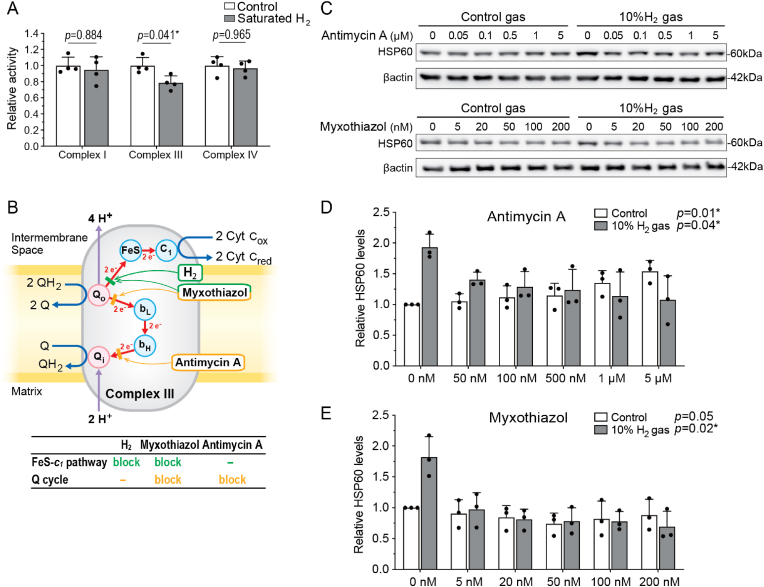
**H_2_ decreased the ETC complex III activity by blocking the FeS-*c*_*1*_ pathway. (A)** Mitochondrial ETC complex activities of isolated mouse liver mitochondria exposed to H_2_-saturated reaction buffer in 2–3 min. *P*-values by two-way ANOVA with Sidak's posthoc test are indicated (*n* = 4 mice each). ∗*P* < 0.05. **(B)** The Q cycle is blocked by both antimycin A and myxothiazol at different sites (yellow bars), whereas the FeS-*c*_*1*_ pathway is blocked by myxothiazol and H_2_ (green bar). Single blockade (antimycin A and H_2_) preserves the responsiveness to UPR^mt^, whereas double blockades (myxothiazol) abolish the responsiveness. Q, ubiquinone. FeS, RISP. **(C, D, E)** Representative Western blotting **(C)** and quantification of HSP60 **(D, E)** in AML12 cells exposed to 10 % H_2_ or control gas in the presence of complex III inhibitors, antimycin A **(C, D)** or myxothiazol **(C, E)**, for 12 h. *P*-values by Jonckheere-Terpstra trend test are indicated (*n* = 3 culture dishes each). The Jonckheere-Terpstra trend test examines whether the change of values is monophasic or not, and gives a single *p*-value for each condition. Statistical significance is indicated by an asterisk.

We next examined the effects of H_2_ on the activities of mitochondrial ETC complexes I, III, and IV, which together constitute the major pathway to generate ATP from NADH. We found that exposing mitochondria isolated from wild-type C57BL6/N mouse liver to H_2_-rich buffer for as little as 2 min suppressed the enzymatic activity of ETC complex III to 78.5 %; however, it did not reduce the activity of complexes I or IV (Fig. 3A). Thus, H_2_ appeared to selectively suppress a critical subunit of ETC complex III.

### 3.4 H_2_ modulates electron flow in complex III in the FeS-c_1_ pathway

To dissect the effects of H_2_ on ETC complex III, we added variable concentrations of complex III inhibitors, antimycin A and myxothiazol, to AML12 cells for 12 h, and evaluated the induction of UPR^mt^ by H_2_. As shown in Fig. 3B, antimycin A blocks electron flow at the Qi site (cytochrome b_H_ → ubiquinone, Q) [14], whereas myxothiazol blocks electron flow at the Qo site (ubiquinol, QH_2_ → cytochrome b_L_) and thereby also electron flow into the FeS-*c*_*1*_ pathway [15] resulting in a more complete blockage.

In control gas-treated AML12 cells, antimycin A induced UPR^mt^ in 12 h in a dose-dependent manner (Fig. 3CD), while myxothiazol failed to do so over the same time course (Fig. 3CE). Exposure to H_2_ gas alone markedly induced UPR^mt^ (Fig. 3C–E). However, combining H_2_ with antimycin A prevented UPR^mt^ induction (Fig. 3CD). This result mirrored the effects of myxothiazol, suggesting that H_2_ may modulate electron transfer into the FeS- *c*_*1*_ pathway. Partial inhibition of electron transfer by H_2_ could disrupt electron flow and semiquinone radical formation, while the combination with antimycin A completely blocks electron transfer, thereby preventing semiquinone radical formation and the induction of UPR^mt^. Thus, the target of H_2_ is likely to be in the FeS-*c*_*1*_ pathway.

### 3.5 H_2_ targets and decreases levels of the Rieske iron-sulfur protein (RISP)

**Fig. 4:**
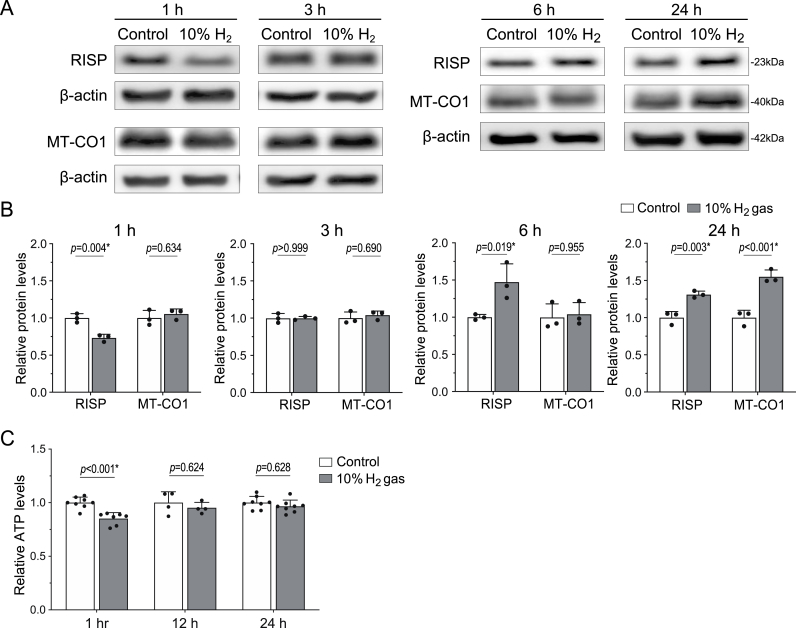
**H_2_ decreased RISP in 1 h and increased it at 6 h and 24 h in AML12 cells. (A, B)** Representative Western blotting **(A)** and quantification **(B)** of nuclear DNA-encoded RISP and mitochondrial DNA-encoded MT-CO1 in AML12 cells cultured under 10 % H_2_ or control gas for 1, 3, 6, and 24 h. *P*-values by two-way ANOVA with Sidak's posthoc test are indicated (*n* = 3 culture dishes). ∗*P* < 0.05. **(C)** ATP levels of AML12 cells cultured under 10 % H_2_ or control gas for 1, 12, and 24 h. *P*-values by two-way repeated measures ANOVA with Sidak's posthoc test (*n* = 7, 4, and 8 culture dishes each at 1, 12, and 24 h, respectively). Statistical significance is indicated by an asterisk.

Hydrogenases that directly react with H_2_ as substrate contain an iron-sulfur cluster (Fe–S cluster) [16]. RISP encoded by *UQCRFS1* is the only component in complex III containing an iron-sulfur cluster, and directly accepts electrons at the Q_0_ site in the FeS-*c*_*1*_ pathway (Fig. 3B). We thus examined the effects of H_2_ on RISP and found that exposure to H_2_ gas for 1 h decreased RISP to 73.3 % in AML12 cells (Fig. 4AB). To explore other potential hydrogen targets, we examined the amounts of representative mitochondrial oxidative phosphorylation complex proteins at 1 h of H_2_ treatment, but no statistical difference was observed in these proteins (Supplementary Fig. S2). We also examined whether RISP degradation was mediated by reduced reactive oxygen species (ROS), such as hydroxyl radicals. To this end, AML12 cells were treated with 2 mM NAC for 18 h to reduce ROS and cultured with 10 % H_2_ for 1 h. NAC marginally reduced the amount of RISP without statistical significance. H_2_ still reduced the amount of RISP even in the presence of NAC, confirming that lowering ROS is not a mediator of H_2_-induced reduction of RISP (Fig. S3) (Supplementary Fig. S3). As NAC failed to cancel the effect of H_2_, the decrease of RISP was unlikely to be medicated by H_2_-mediated reduction of free radicals, if any. The RISP level resumed to baseline at 3 h and increased to 147.4 % at 6 h and 131.2 % at 24 h. In accordance with the decrease of RISP, H_2_ gas decreased the ATP level to 85.1 % at 1 h, but resumed it to the basal level at 12 and 24 h (Fig. 4C). In contrast, the level of cytochrome *c* oxidase subunit I in complex IV that is encoded by *MT-CO1* on mitochondrial DNA remained unchanged up to 6 h but was increased to 155.4 % at 24 h (Fig. 4AB). As mitonuclear protein imbalance is one of the major causes inducing UPR^mt^ [17], transient reduction of RISP at 1 h likely initiated UPR^mt^, which subsequently led to a compensatory increase in both RISP and MT-CO1 in 24 h. We also examined the effects of H_2_ in other cell lines and found that H_2_ treatment for 1 h decreased RISP in HT1080 and HeLa cells (Supplementary Fig. S4).

### 3.6 Mitochondrial Lon peptidase 1, LONP1 mediates the H_2_-induced degradation of RISP

**Fig. 5:**
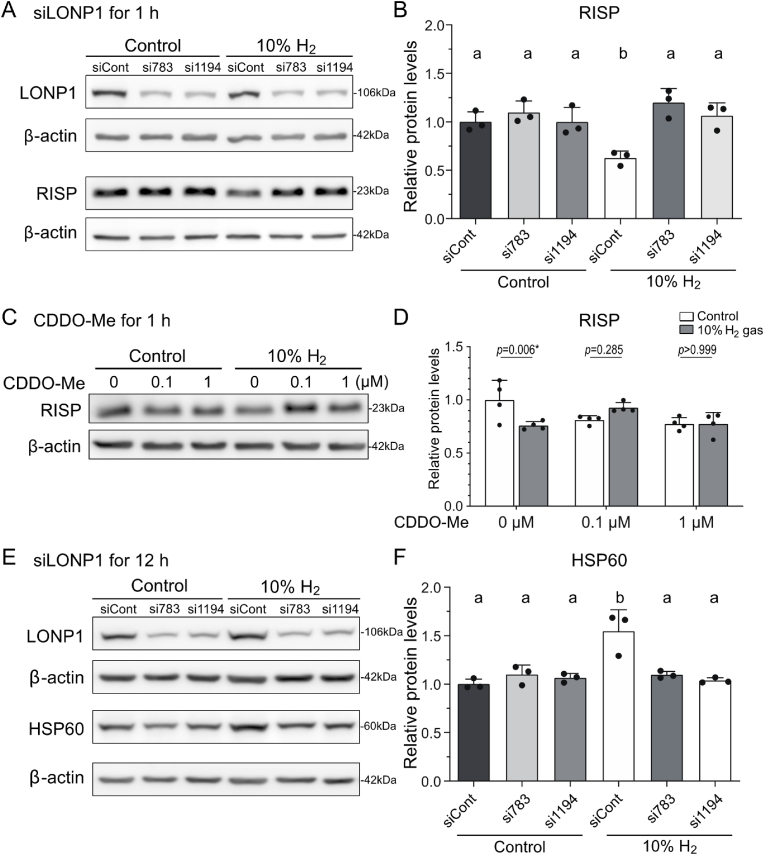
**Inhibition of LONP1 by knockdown or a specific inhibitor cancelled the reduction of RISP by H_2_ in AML12 cells.** Representative Western blotting **(A, C, E)** and quantification **(B, D, F)** of RISP **(A, B, C, D)** and HSP60 **(E, F)** in AML12 cells cultured under 10 % H_2_ or control gas, while LONP1 was knocked down by si783 or si1194 **(A, B, E, F)** or inhibited by CDDO-Me **(C, S)** (*n* = 3, 4, and 3 culture dishes for **B**, **D**, and **F**, respectively). **(B, F)***P* < 0.05 by one-way ANOVA with Tukey's posthoc test is indicated by ‘a’ and ‘b’. **(D)***P*-values by two-way ANOVA with Sidak's posthoc test (asterisk indicates statistical significance).

Mitochondrial Lon peptidase 1 encoded by *LONP1* is a major mitochondrial protease that selectively degrades misfolded, unassembled, or damaged polypeptides in mitochondria, and plays a substantial role in the induction of UPR^mt^ [18]. We asked whether LONP1 was involved in the H_2_-mediated degradation of RISP, and found that knockdown of LONP1 nullified the effects of H_2_ on the decrease of RISP in 1 h (Fig. 5AB). Similarly, a LONP1 inhibitor, CDDO-Me, cancelled the effects of H_2_ on the decrease of RISP in 1 h (Fig. 5CD). Thus, H_2_ triggered LONP1-mediated degradation of RISP. In addition, LONP1 knockdown cancelled the increase of HSP60 expression by H_2_ gas in 12 h (Fig. 5EF), indicating that a conformational change of RISP induced by H_2_ is a key to trigger LONP1 and UPR^mt^. The selective degradation of RISP following LONP1 activation, which specifically targets misfolded/unfolded/damaged mitochondrial proteins, suggests that RISP is a primary target of H_2_.

## 4 Discussion

Specific suppression of ETC complex III by H_2_ and the presence of an iron-sulfur cluster in hydrogenases in evolution prompted us to examine the effects of H_2_ on the only iron-sulfur cluster-bearing molecule in complex III, RISP. We indeed found that H_2_ primarily targets RISP, and initiates its LONP1-mediated degradation. Notably, RISP is unique among mammalian iron-sulfur proteins in its coordination of the [2Fe–2S] cluster by two histidines, which elevate the cluster's redox potential compared to the cysteine-only coordination seen in other iron-sulfur proteins [19]. This unique configuration may underlie RISP's favorable interaction with H_2_, in contrast to other cysteine-coordinated iron-sulfur-bearing proteins that appear unresponsive to H_2_ [20].

The amount of RISP should be determined by a balance between LONP1-mediated degradation and its subsequent compensatory UPR^mt^-mediated induction. In the presence of H_2_, LONP1 continues to degrade RISP, initiating a transient mitonuclear protein imbalance that triggers UPR^mt^. This compensatory response upregulates RISP synthesis, leading to a recovery and eventual overshoot of RISP levels at later time points, as observed at 6 and 24 h (Fig. 4B). We previously showed that ingestion of H_2_ water or intermittent inhalation of H_2_ gas, but not continuous inhalation of H_2_ gas, ameliorated a rat model of Parkinson's disease [21]. These results may be accounted for by the discontinuation of RISP degradation by intermittent H_2_ inhalation or drinking H_2_ water. This suggests that intermittent exposure to H_2_ is sufficient to optimizes the balance between stress induction and adaptive recovery, a hallmark of hormesis.

We found that the induction of UPR^mt^ was associated with a biphasic modulation of mitochondrial superoxide production, membrane potential, ATP levels, and the enzymatic activity of ETC Complex III, suggesting a tightly regulated hormetic response. The initial suppression of superoxide and membrane potential within 10 min of H_2_ exposure (Fig. 1AB) likely reflects transient inhibition of electron flow through Complex III, as evidenced by the reduction of its enzymatic activity to 78.5 % observed in isolated mitochondria (Fig. 3A). This disruption correlated with a decrease in ATP levels to 85.1 % at 1 h (Fig. 4C), indicative of reduced mitochondrial activity. However, over the subsequent hours, superoxide production and membrane potential increased significantly (Fig. 1AB), corresponding to the restoration of ATP levels to baseline by 12 and 24 h (Fig. 4C). This compensatory response likely reflects an adaptive enhancement of mitochondrial function driven by mitohormesis. While the magnitude of UPR^mt^ induction in AML12 cells by H_2_ was less pronounced compared to studies using chemical stress inducers or genetic engineering to provoke mitonuclear imbalance [11], it is important to note that these experimental conditions exceed physiological feasibility and relevance. In contrast, even high doses/concentrations of H_2_ can be readily applied to humans without adverse effects [22].

The biphasic mitochondrial dynamics observed in this study reconcile seemingly contradictory findings in previous reports on markers of reactive oxygen species (ROS) and inflammation. Similar to other small gaseous signaling molecules (NO^•^, CO, H_2_S) [23], H_2_ has been shown to paradoxically increase or decrease various molecules, pathways, and indicators. These include malondialdehyde [24], derivatives of reactive oxygen species [25], superoxide levels [26], 8-hydroxy deoxyguanine [27], Nrf2 [28], NF-κB [28,29], heat shock proteins [30], ATP levels [6,26], mitochondrial membrane potential [31], and ETC complex activity [32], and mitophagy [33], while simultaneously providing therapeutic effects that promote cellular survival under stress conditions. Early mechanistic studies proposed that H_2_ exerted its effects not as a biologically active molecule, but solely through its chemical property of reacting with hydroxyl radicals [6,9]. Thus, these studies fail to account for H_2_'s temporally dynamic and seemingly paradoxical effects on redox and inflammatory pathways. In contrast, our findings reveal that H_2_ is a biologically active agent, mediating its biphasic response through the selective modulation of Complex III, altering electron transport to trigger mitochondrial signaling and adaptive stress pathways, such as UPR^mt^

Mitochondria are descendants of ancient hydrogenase that relied on H_2_ for their energy systems and redox regulation long before oxygen dominated Earth's atmosphere [34]. In this study, we demonstrate that molecular hydrogen, a gas integral to early life forms, specifically targets RISP within mitochondrial ETC Complex III. This hormetic interaction links two primordial elements, mitochondria and hydrogen, and redefines H_2_ from a biologically inert molecule to a biologically active signaling molecule. Given the growing body of clinical research on H_2_, elucidating its precise mechanism of action provides critical insights that can guide the design and interpretation of clinical trial protocols, optimizing its therapeutic potential.

Our study has the following limitation. Although we showed that H_2_ induced LONP1-mediated degradation of RISP, we did not examine the exact conformational changes in RISP induced by H_2_ or how LONP1 recognized H_2_-exposed RISP as a target for degradation.

## 5 Conclusion

We showed that H_2_ primarily targets RISP in mitochondrial ETC complex III. This targeting leads to (i) LONP1-mediated degradation of RISP, (ii) initial suppression of mitochondrial ETC activity, followed by (iii) activation of mitochondrial ETC activity via induction of UPR^mt^ by mitonuclear protein imbalance and increased reactive oxygen species. The elucidated mechanism readily accounts for the temporally diverse and ostensibly paradoxical effects of H_2_ on redox and inflammatory markers. Given the growing body of clinical research on H_2_, elucidating its precise mechanism of action provides critical insights that can guide the design and interpretation of future clinical studies to evaluate its effectiveness and optimize its therapeutic potential.

## CRediT authorship contribution statement

**Shuto Negishi:** Investigation, Writing – original draft, Methodology. **Mikako Ito:** Conceptualization, Formal analysis, Project administration, Writing – original draft, Investigation, Methodology, Funding acquisition. **Tomoya Hasegawa:** Investigation. **Hikaru Otake:** Investigation. **Bisei Ohkawara:** Methodology, Project administration. **Akio Masuda:** Methodology, Project administration. **Hiroyuki Mino:** Methodology, Supervision. **Tyler W. LeBaron:** Conceptualization, Supervision, Writing – review & editing. **Kinji Ohno:** Conceptualization, Supervision, Writing – review & editing, Funding acquisition.

## Data and materials availability

The data that support the findings of this study are available from the corresponding authors upon reasonable request.

## Funding

This work was supported by Grants-in-Aid from the 10.13039/100009619Japan Agency for Medical Research and Development (JP23ek0109678); the 10.13039/501100001691Japan Society for the Promotion of Science (JP23H02794, JP23K18273, JP21H02476, JP22K19269, and JP23K06412); the 10.13039/501100003478Ministry of Health, Labour and Welfare of Japan (23FC1014); and the 10.13039/501100009438National Center of Neurology and Psychiatry (5-6).

## Declaration of competing interest

The authors declare no conflict of interest.
