# Supplementary material for: The Rieske iron-sulfur protein is a primary target of molecular hydrogen
Source: Redox Biol. 2025 Nov 27;88:103952. doi: 10.1016/j.redox.2025.103952 (PMC12719095; doi:10.1016/j.redox.2025.103952)
Supplement: Multimedia component 1 [file mmc1.docx]

**a**

**Supplementary Fig. S1**. **H_2_ does not affect mitochondrial fission- and fusion-related proteins in AML12 cells.** AML12 cells were exposed to 10% H_2_ or control gas for 6 h. Representative Western blotting **(A)** and quantification **(B)** of fission-related proteins (Mfn1/2 and Opa1) and fusion-related proteins (Drp1 and p-Drp1 at Ser616). **(C)** Relative ratio of p-Drp1 at Ser616 to total Drp1. *P*-values by two-way ANOVA with Sidak’s posthoc test **(B)** and Student’s *t*-test **(C)** (*n* = 4 culture dishes each). There was no statistical difference.

**Supplementary Fig. S2**. **H_2_ does not affect the levels of oxidative phosphorylation complex proteins other than RISP in AML12 cells.** AML12 cells were exposed to 10% H_2_ or control gas for 1 h. Representative Western blotting **(A)** and quantification **(B)** of oxidative phosphorylation complex proteins (ATP5F1A, UQCRC2, MT-CO1, SDHB, NDUFB8, and RISP). *P*-values by two-way ANOVA with Sidak’s posthoc test (left panel) and Student’s *t*-test (right panel) (*n* = 3 culture dishes each). Statistical significance is indicated by an asterisk.


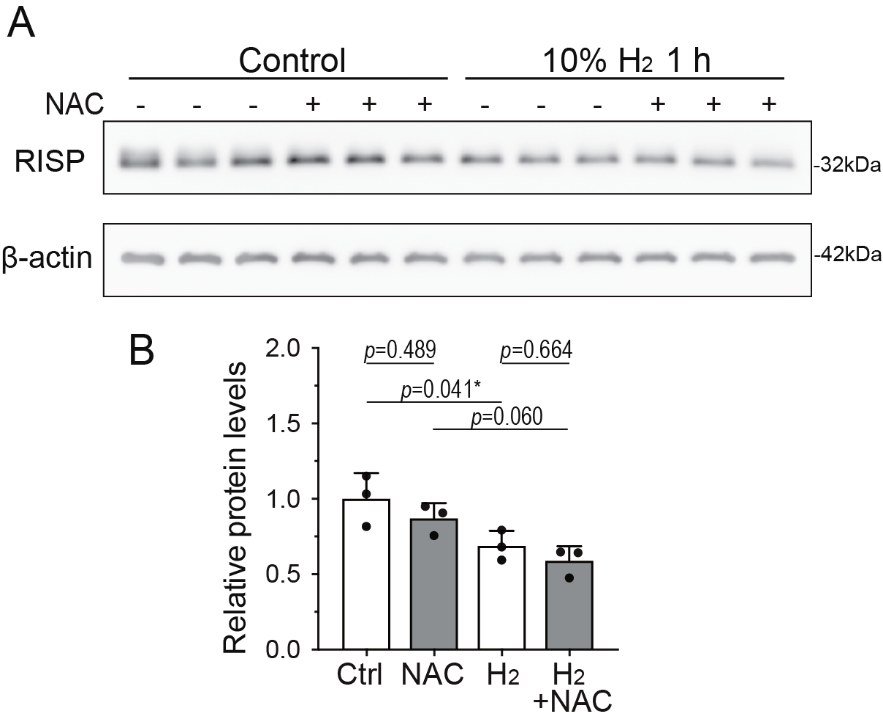


**Supplementary Fig. S3.** The effect of 2 mM N-acetylcysteine (NAC) treatment for 18 h on the H_2_-mediated reduction of RISP in AML12 cells. Cells were exposed to 10% H_2_ gas for 1 h before protein extraction. *P* values by two-way-ANOVA with Tukey’s posthoc test (*n* = 3 culture dishes each). Statistical significance is indicated by an asterisk.

**Supplementary Fig. S4**. **H_2_ reduce RISP levels in HT1080 and HeLa cells.** HT1080 and HeLa cells were exposed to 10% H_2_ or control gas for 1 h. Representative Western blotting **(A)** and quantification **(B)** of RISP protein. *P*-values by two-way ANOVA with Sidak’s posthoc test are indicated (*n* = 4 or 3 culture dishes each). Statistical significance is indicated by an asterisk.

**Supplementary Table S1. Antibodies for Western blotting**

| **Name** | **Manufacturer** | **Cat. No.** | **Dilution** |
| --- | --- | --- | --- |
| PKR | Santa Cruz Biotechnology | sc-6282 | 1:1000 |
| p-eIF2a | Santa Cruz Biotechnology | 9721 | 1:1000 |
| eIF2a | Cell Signaling Technology | 5324 | 1:500 |
| HSP60 | Cell Signaling Technology | 4870 | 1:1000 |
| ATF5 | Abcam | ab184923 | 1:1000 |
| UQCRFS1 | Abcam | ab14746 | 1:1000 |
| UQCRC2 | Santa Cruz Biotechnology | sc-292924 | 1:1000 |
| MT-CO1 | Abcam | ab14705 | 1:1000 |
| LONP1 | Sigma | HPA002192 | 1:1000 |
| β-actin | Santa Cruz Biotechnology | sc-47778 | 1:1000 |
